# Anakinra pharmacokinetics in children and adolescents with systemic-onset juvenile idiopathic arthritis and autoinflammatory syndromes

**DOI:** 10.1186/2050-6511-14-40

**Published:** 2013-08-05

**Authors:** Saik Urien, Christophe Bardin, Brigitte Bader-Meunier, Richard Mouy, Sandrine Compeyrot-Lacassagne, Franz Foissac, Benoît Florkin, Carine Wouters, Bénédicte Neven, Jean-Marc Treluyer, Pierre Quartier

**Affiliations:** 1 CIC-0901 Inserm Necker-Cochin (Assistance Publique-Hopitaux de Paris), Paris EA-3620, France; 2 Université Paris Descartes Sorbonne Paris Cité et Institut IMAGINE, Paris, France; 3 Pharmacologie, Hôpital Cochin, Assistance Publique-Hopitaux de Paris, Paris, France; 4 Unité d’Immuno-Hématologie et Rhumatologie pédiatriques, Hôpital Necker-Enfants Malades, Assistance Publique-Hopitaux de Paris, Paris, France

## Abstract

**Background:**

Anakinra pharmacokinetics and pharmacodynamics were investigated in children and adolescents treated for systemic-onset juvenile idiopathic arthritis (SJIA) and autoinflammatory syndromes.

**Methods:**

Anakinra was given subcutaneously at doses between 2 and 10 mg/kg (maximum 100 mg) per day. Anakinra concentrations were recorded in patients, as well as C-reactive protein (CRP) levels, on different occasions. The data were fitted to a pharmacokinetic-pharmacodynamic model via a population approach using Monolix.

**Results:**

A total of 87 children and adolescents, 8 months to 21 years old, were available for pharmacokinetic evaluation. A one compartment model with linear absorption and elimination described the pharmacokinetics. Taking into account bodyweight to explain variations in apparent clearance (CL/F) and distribution volume (V/F) significantly reduced the associated between-subject and between-occasion variabilities. The final estimates were 6.24 L/h/70 kg and 65.2 L/70 kg for CL/F and V/F respectively. A mixture pharmacodynamic model described the CRP level change during anakinra treatment for the SJIA patients with 2 subpopulations, patients with high baseline and large CRP decrease and patients with low baseline and small CRP decrease followed by a re-increase in CRP levels. There was no significant effect of the combined anti-inflammatory treatment. The proportion of patients for which the development of a resistance to treatment was significant was 62% and the corresponding time was approximately 60 days.

**Conclusions:**

Based on effects in SJIA, a prospective dosage adjustment was proposed based on a 0.4 mg/L Css target in order to obtain a CRP decrease to 10 mg/L or below.

## Background

Anakinra, a recombinant nonglycosylated homolog of human IL-1 receptor antagonist, competitively inhibits the binding of IL-1α and IL-1β to the IL-1 receptor and thus inhibits the effects of this pro-inflammatory cytokine. Il-1 blockade using selective IL-1β blockade (canakinumab) or IL-1 α and β blockade (anakinra, rilonacept) has proven efficacy in cryopyrin-associated periodic syndromes (CAPS) [1] and more recently in systemic-onset juvenile idiopathic arthritis (SJIA) [2-5]. In patients with CAPS, anakinra has been shown to induce clinical remission in most cases, decrease the inflammatory markers including C-reactive protein (CRP) and improve patient’s quality of life on the long term [1,6]. In the first randomized placebo-controlled trial published with anakinra in SJAI, the number of active joints, CRP and the physician global disease activity assessment using a visual analalog scale (VAS) were significantly decreased in the anakinra group compared to the placebo group at one month. However, some patients eventually experienced a disease flare and the authors hypothesised that suboptimal anakinra dosage might be partly responsible for a lack of sustained efficacy, in low-weight children [3].

The aim of the present study was to investigate anakinra pharmacokinetics in children and young adult patients with SJIA and autoinflammatory conditions. The effects of anakinra on CRP was also modelled in relation to the anakinra pharmacokinetics in children with SJIA.

## Methods

### Patients

Following IRB (CPP Paris V) approval and patients and/or parents (for children) written informed consent, pharmacokinetic data were obtained from a phase IIB trial testing anakinra in SJIA patients (ANAJIS)(5) and from patients subsequently treated for diverse autoinflammatory conditions. In ANAJIS trial all the patients received anakinra once-a-day at the dose of 2 mg/kg subcutaneously, maximum 100 mg. In patients with autoinflammatory syndromes treated afterwards, anakinra was given at doses ranging between 2 and 10 mg/kg daily (the highest doses were in low-weight CAPS patients who had failed to respond to lower doses). In the patients who took part to ANAJIS trial, CRP was recorded at each visit and retained for the pharmacodynamic modeling.

### Anakinra plasma determination

Anakinra plasma determinations were performed on blood taken at one or repeated occasions depending on the study group. Whole blood samples were collected into tubes containing heparin. Plasma was separated immediatly after sampling and frozen at −20°C. Concentrations of anakinra in plasma samples were determined using the antibody (Ab) ELISA purchased from R&D Systems (Minneapolis, Minnesota, USA). Briefly, samples and quality controls were diluted with buffered animal serum and added to a microtiter plate which have been pre-coated with a monoclonal antibody specific for IL-1ra. An enzyme-linked polyclonal antibody specific for IL-1ra (horseadish peroxydase) was added to the wells. Following a wash, a substrate solution was added for color development. Reaction was stopped with sulfuric acid. Optical density was determined using a microplate reader set to 450 nm. Anakinra concentration were calculated for each sample by log-log curve fitting of the plate standards dilutions. The lower limit of quantification (LOQ) for anakinra concentrations in plasma samples was 40 ng/mL.

### Pharmacokinetic modelling

Pharmacokinetic data was ascribed to an open one-or two-compartment models with linear absorption. Zero-order absorption and absorption with transit compartments models, as well as the possibility of non-linear elimination, were also considered.

### Pharmacodynamic modelling

The CRP levels as a function of time and drug treatment were ascribed to a indirect response model. In this model, anakinra is thought to inhibit the response production rate, k_TR_*R0 (transit time rate constant multiplied by response at baseline). The model equation was then

(1) $$\mathrm{dR}/\mathrm{dt}=k_{\mathrm{TR}}*R_{0}*\left[1-C/\left(C_{50}+C\right)\right]-k_{\mathrm{TR}}*R$$

where R, C_50_ and C stand for the pharmacodynamic response, 50% inhibitor concentration and drug concentration. When the drug treatment starts, the system is at equilibrium (stable disease) what is defined by the baseline parameter, R = R_0_.

Some individual time-courses showed that there was a loss of drug effect during the treatment time. To take this into account, an empirical resistance function was as a function of time was defined

$$\mathrm{Resis}=\exp\left(-k_{\mathrm{RESI}}t\right)$$

where k_RESI_ is a time rate constant of resistance appearance. Then the response model becomes

(2) $$\mathrm{dR}/\mathrm{dt}=k_{\mathrm{TR}}*R_{0}*\left[1–\mathrm{Resis}\;C/\left(C_{50}+C\right)\right]\;-\;k_{\mathrm{TR}}*R$$

Therefore, using a mixture model, the patients were ascribed to either equation (1) or (2). The CRP levels were analysed in the 22 SJIA patients from the ANAJIS trial.

### Population pharmacokinetic and pharmacodynamic analysis

Pharmacokinetic and pharmacodynamic data were analysed using the nonlinear mixed effect modelling software program Monolix version 3.2 (http://www.lixoft.com/) [7]. Pharmacodynamic data were obtained from the 22 ANAJIS trial patients: the CRP concentrations were log_10_–transformed to take into account the wide range of observed data during the analysis. The data were analysed sequentially; the pharmacokinetic estimates were fixed for the pharmacodynamic analysis. Parameters were estimated by computing the maximum likelihood estimator of the parameters without any approximation of the model (no linearization) using the stochastic approximation expectation maximization (SAEM) algorithm combined to a MCMC (Markov Chain Monte Carlo) procedure. The number of MCMC chains was allowed to vary in order to obtain a nice and reliable convergence of the SAEM algorithm. Additive and proportional error models were used to describe the residual variability (ϵ) for the pharmacokinetic and pharmacodynamic data respectively, and the between-subject or between occasion variabilities (η or φ) were generally ascribed to an exponential model, except specific indication. The likelihood ratio test (LRT) including the log-likelihood, the Akaike information criterion (AIC) and the bayesian information criterion (BIC) was used to test different hypotheses regarding the final model, covariate effect on structural parameter(s), residual variability model (proportional versus proportional plus additive error model), structure of the variance-covariance matrix for the BSV parameters. The normalised prediction distribution errors (NPDE) metrics [8] were used used as a main diagnostic tool to evaluate the final model and were directly computed by Monolix. Diagnostic graphics and other statistics were obtained by using RfN (http://wfn.sourceforge.net/) with the R program [9].

## Results

### Population pharmacokinetic modeling

**Figure 1:**
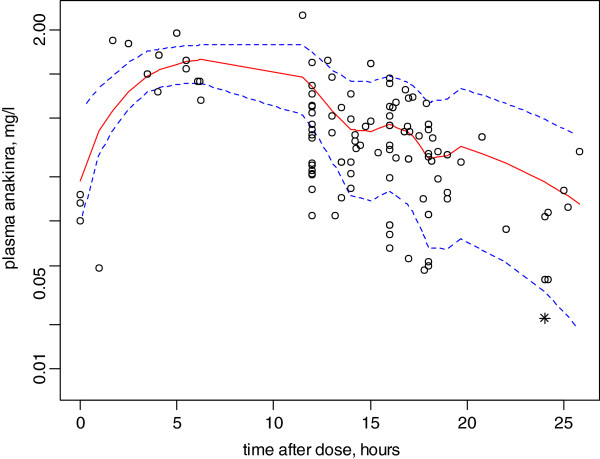
**Anakinra concentration-time courses for once daily administrations.** Solid and dashed lines, median and 5^th^/95^th^ percentiles of the final model predictions.

A total of 87 children (32 girls, 52 boys) with 148 anakinra concentrations were available for pharmacokinetic evaluation, four concentrations were observed below the limit of quantification (BLQ) and coded as left censored data for the analysis. The distribution of sampling times can be observed in Figure 1.

The 22 SJIA patients were 2.26 – 16.8 years old (median 7.6) weighing 10 – 83 kg (median 21). The other patients were 0.73 – 21 years old (median 8) weighing 4.3 – 60 kg (median 21). These included 20 patients with CAPS (14 with the chronic, inflammatory, neurologic, cutaneous and articular (CINCA) syndrome / neonatal onset multisystem inflammatory disease (NOMID) and 6 with Muckle-Wells syndrome), mevalonate kinase deficiency (n = 3), TNF-receptor associated periodic syndromes (n = 2), familial mediterranean fever (n = 1) and patients with genetically undetermined autoinflammatory conditions.

A one-compartment with linear absorption and elimination model adequately described the data. Other candidate models included one-compartment with non-linear elimination and two-compartment models. The parameters of the model were Ka, V/F and CL/F, respectively the absorption rate, the apparent volume of distribution and clearance, F being the unknown bioavailability. The statistical model included a between-subject variability for CL/F, η_CL_, and a between-occasion variability for V/F, γ_V/F_. The residual variability was described as an additive component.

The only covariates that influenced the pharmacokinetic parameters were age and bodyweight (BW). CL/F and V/F were then related to BW using an allometric function with estimated power exponents. This decreased the CL/F and V/F variabilities from 0.41 to 0.28 and from 1.34 to 0.475 and the AIC and BIC criteria by more than 15 units. This BW effect finally removed the effect of age. No other covariate effect could be identified, gender or combined use of corticoids or anti-inflammatory drugs, AINS. The final models for CL/F and V/F were then

$$\begin{array}{c} \mathrm{CL}/F=0.847\times BW^{0.47}\\ V/F=2.581\times BW^{0.76} \end{array}$$

Figure 1 depicts anakinra observed time-courses and the median and 5^th^/95^th^ percentiles of the model predictions. Table 1 summarises the final population pharmacokinetic estimates. As shown, all parameters were well estimated with low relative standard errors.

**Table 1:** Parameter estimates of the final anakinra population pharmacokinetic model in 87 pediatric patients

**Parameter**	**Estimate**	**Relative standard error (%)**
CL/F (L h^-1^ 70 kg^-1^)	6.24	8
β_CL_, TV(CL)∙(BW/70)^βCL^	0.47	14
V/F (L)	65.2	12
β_V_, TV(V)∙(BW/70)^βV^	0.76	16
Ka (h^-1^)	0.38	19
η_CL/F_	0.28	15
γ_V/F_	0.47	17
ϵ, mg/L	0.072	10

Key: *CL/F*, apparent elimination clearance; *V/F*, apparent volume of distribution; *Ka*, absorption rate constant; *F*, unknown bioavailability; *TV()*, typical value for the mean covariate value; *β*, covariate effect parameter; *η*, between-subject variability; *γ*, between occasion variability; *ϵ*, constant residual variability; *BW*, bodyweight (CL/F and V/F estimates are normalized to a 70 kg BW).

### Pharmacodynamic modeling

**Figure 2:**
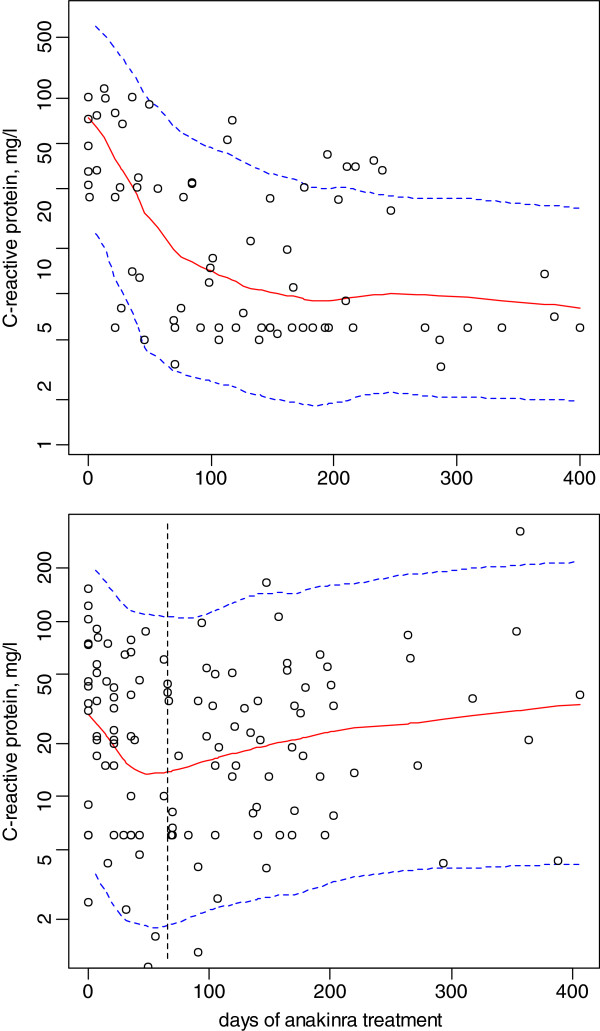
**Effect of anakinra on the C-reactive concentration-time courses during the ANAJIS trial.** The top and bottom plots stand for the 2 response groups : top, responders; bottom, patients with a delayed “resistance” to treatment. Solid and dashed lines, median and 5^th^/95^th^ percentiles of the final model predictions.

An indirect response model assuming that the production rate of the inflammatory process is inhibited by anakinra concentrations described well the CRP time-courses in the 22 ANAJIS trial patients (195 observations were available). The C_50_ parameter was related to the mean steady-state anakinra concentration (Css), allowing the determination of the corresponding dosage to obtain for example 90% of the maximal effect (C_90_ ~ 10 x C_50_), i.e., dose rate (mg/h) = CL/F*C_90_. A mixture model with 2 subpopulations, patients with high baseline and large CRP decrease and patients with low baseline and small CRP decrease followed by a slow re-increase in baseline levels (delayed “resistance” to treatment), was finally retained. The corresponding observed baselines (median [range]), 120 [44, 230] and 43 [2.5, 152] mg/L, were significantly different (p = 0.02, Kruskal-Wallis test). However it was impossible to estimate a clear cut-off value between the 2 groups, probably because of the small sample size. There was no significant effect of the combined anti-inflammatory treatment. The proportion of patients for which the development of a “resistance” to treatment was significant was 62% and the corresponding time was approximately 60 days. Table 2 summarizes the results and Figure 2 depicts the CRP observed time-courses and the median and 5^th^/95^th^ percentiles of the model predictions.

**Table 2:** Parameter estimates of the anakinra effect on c-reactive protein concentrations in 22 SJIA patients (RESP = responders and RESI = patients with onset of “resistance” to treatment)

**Parameter**	**Estimate**	**Relative standard error (%)**
Baseline (mg/L)		
RESP	141	27
RESI	37.9	29
k_TR_ (day^-1^)	0.042	27
C_50_ (mg/L)	0.03	37
k_RESI_ (day^-1^)	0.0048	0.0018
Proportion of RESP	0.37	31
η_BASELINE.RESI_	0.79	24
η_KTR_	081	25
ϵ, mg/L (*)	0.39	6

Key: *Baseline*, CRP level before treatment; *k*_*TR*_, transit time rate constant; *C*_*50*_, anakinra concentration that induces a 50% decrease of CRP level; *k*_*RESI*_, time rate constant of resistance appearance; *η*, between-subject variability; *ϵ*, constant residual variability, *log-additive model.

### Evaluation and validation

Figures 1 and 2 show that the average prediction matches the observed concentrations or CRP time courses and that the variability is reasonably estimated. Moreover, the NPDE residuals corresponding to these modellings also validated these population models, the mean and variance were not significantly different from 0 and 1 and the distribution did not differ from normality.

### Dosage simulations

**Figure 3:**
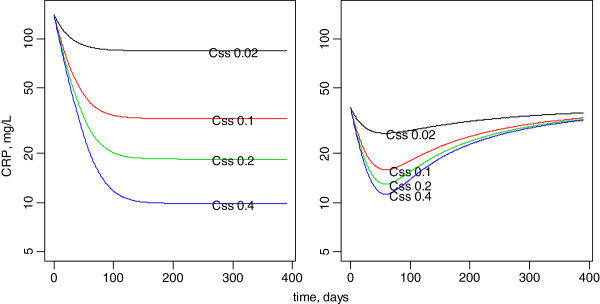
**Model-predicted effect of anakinra on the C-reactive concentration-time courses assuming 0.02 to 0.40 mg/L mean steady-state anakinra concentrations in the 2 subgroups of patients.** Left, high base level with large CRP decrease. right, moderate base CRP with initial decrease followed by a re-increase in CRP concentrations.

**Figure 4:**
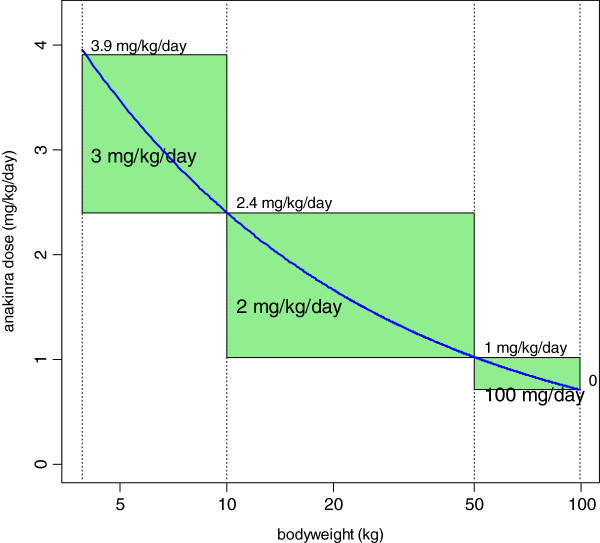
**Daily dose of anakinra (mg/kg, thick curve) as a function of bodyweight in order to reach the mean anakinra steady-state concentration of 0.4 mg/L.** The 0.4 mg/L target corresponds to a maximal effect on the CRP biological marker of inflammation. Bold text in rectangles defines possible mean dosage recommendation for 3 bodyweight ranges. Text on top side stands for the maximal dosage in the corresponding bodyweight range.

Figure 3 shows the model-predicted CRP time-courses for different Css values. Clearly, the target Css is 0.4 mg/L in order to obtain a CRP decrease to 10 mg/L or below. Accordingly, the anakinra dosage as a function of bodyweight can be deduced as shown in Figure 4.

## Discussion

This is the first pharmacokinetic study of anakinra in children. In pediatric patients with SJIA and diverse autoinflammatory conditions, the pharmacokinetics of anakinra following sub-cutaneous administration was satisfactorily described by a one compartment model with 1^st^ order absorption. Bodyweight was identified as the sole significant covariate that could finally explain a significant part of the variability and this BW effect was modeled via an allometric scaling of CL/F and V/F. The allometric power values are typically 1 and 0.75 for BW effect on V and CL respectively [10]. Because our estimates were not so close to these theoretical values and because anakinra is a 153 amino acids peptide chain and somewhat different from standard drugs, the estimated power effects were retained in the final pharmacokinetic model. The improvement of the predictive performance of the model (observed vs. predicted data, not shown) as well as a decrease in the CL/F and V/F variabilities validated this model.

Assuming that anakinra inhibits the production of the inflammatory marker CRP via an “Emax” model allowed a satisfactory description of the CRP time-course during anakinra treatment in SJIA patients. As seen in Figure 2, this was monitored and modelled up to approximately 400 days of treatment. The C_50_ estimate was low (0.03 mg/L) and nearly all the observed concentrations were above this limit. According to the predicted time-course effects of different mean Css values (Figure 3), a Css value of 0.4 mg/L was retained to obtain the maximal effect. The dosages per 24 h that produce this 0.4 mg/L Css are depicted in Figure 4. Therefore, the actual dosage of 2 mg/kg/day in SJIA patients was appropriate in the 10 – 50 kg BW range children, but not in low weight or early age children, BW < 10 kg, for which the efficient mean dose would be 3 mg/kg/day. Also, the oldest chilgren, > 50 kg BW, could have received a flat dose of 100 mg/day, similar to the adult dosage.

## Conclusions

Bodyweight significantly influenced the pharmacokinetics of anakinra. An allometric BW scaling of apparent clearance and distribution volume was sufficient to describe the variation of anakinra pharmacokinetics in these 87 pediatric patients weighing from 4 to 80 kg and 9 months to 21 years old. The effect on the C-reactive protein was adequately described by a turn-over model and allowed to derive some concentration-dose guidelines for SJIA disease.

## Competing interests

The authors declare that they have no competing interests.

## Authors’ contributions

SU, JMT and PQ wrotre the paper; SU and FF performed the modelling and analysis of the data; PQ, JMT, BBM, CB designed the research; CB was responsible for the drug assay; BBM, RM, SCL, BF, CW, BN, PQ conducted the research; SU, PQ and JMT had primary responsibility for the final content. All authors read and approved the final content.

## Pre-publication history

The pre-publication history for this paper can be accessed here:

http://www.biomedcentral.com/2050-6511/14/40/prepub
